# Editorial: Mental health in healthcare workers and its associations with psychosocial work conditions

**DOI:** 10.3389/fpubh.2024.1399134

**Published:** 2024-04-03

**Authors:** Juan Jesús García-Iglesias, Murat Yildirim, Juan Gómez-Salgado, Yong-Shian Shawn Goh

**Affiliations:** ^1^Department of Sociology, Social Work and Public Health, Faculty of Labour Sciences, University of Huelva, Huelva, Spain; ^2^Department of Psychology, Faculty of Science and Letters, Agri Ibrahim Cecen University, Agri, Türkiye; ^3^Department of Social and Educational Sciences, Lebanese American University, Beirut, Lebanon; ^4^Escuela de Posgrado, Universidad de Especialidades Espíritu Santo, Guayaquil, Ecuador; ^5^Alice Lee Centre for Nursing Studies, National University of Singapore, Singapore, Singapore

**Keywords:** mental health, health personnel, work condition, psychological distress, burnout, anxiety

Working conditions impact the mental health of healthcare workers (HCWs) and vice versa (1). This may affect safety in the exercise of the activities to be carried out, which could lead to a greater number of accidents at work and the quality of the service provided (2).

The mental health of HCWs is reported to be impaired to a greater or lesser extent in all countries of the world. Each country has its idiosyncrasies, and different levels of care for HCWs are applied within different countries and regions. At the same time, each job is unique and changing (3). Of particular relevance in this regard is the change in many of the working conditions of healthcare providers worldwide in their efforts to combat the effects of the COVID-19 pandemic (4).

The studies included within this Research Topic bring attention to how the mental health of HCWs has been assessed differently across contexts, from East to West.

Only one study was conducted in Africa, where Traoré et al. found that 3 out of 10 HCWs had high-stress levels, mainly due to the risk of being exposed to contamination and being the focus of contamination.

In Europe, the study by Bosma et al. is highlighted. It was carried out in the northern area of the Netherlands, and the total scores for anxiety during the COVID-19 pandemic were similar for HCWs and non-HCWs. This differs from the results reported by van der Noordt et al. during the first year of the pandemic in the same country. On its part, the study by Rypicz et al. showed that the level of education in healthcare correlated with a higher likelihood of experiencing stress and burnout, especially among nurses. In Germany, a study was carried out on healthcare assistants by Schrader et al. where, in March/April 2020, 29.5% of respondents reported feelings of very high or high psychological distress due to concerns about the patient's health, uncertainty about the new disease, work/family balance, and fear of infecting family members with COVID-19. In turn, Echeverria et al. conducted a longitudinal study in Spain (April-May 2020 and September-October 2021) in which 29.5% of the sample felt that their mental health had improved during this time, 47.7% said it had not changed, and 22.7% reported that their mental health had worsened. Meanwhile, in Italy, 46.5% of HCWs working in vaccination centers reported that they had been victims of violence during the vaccination campaign, of which 35.5% of cases may have been related to a possible post-traumatic stress reaction, according to Brunelli et al..

In China, the study by Lv et al. showed that 64.71% of HCWs considered their occupational stress high or very high, with overly intense work as the primary stressor, being the prevalence of anxiety and depression among the HCWs of 45.2 and 41.4%, respectively, such as in the study of Liu et al.. In another sample of HCWs, the prevalence of symptoms of depression and anxiety among primary health care workers was 67.3% and 55.5%, respectively, with stress, social support, and self-efficacy being influential factors, according to Dong et al.. These data align with Cai et al.'s findings in primary care centers, where the burnout rate was 59.87%, influenced by work environment, professional pride, work intensity, and salary. In the study by Huang R. et al., 78.3% of resident physicians had experienced at least one traumatic event, with the rate being higher in subjects aged 26–30 years, females, and those with a higher number of working hours. Working hours were also positively associated with occupational stress in the study by Lu et al.. As expected, the results found by Xue et al. showed that nurses with burnout imagined fewer specific future events, positive events, and events related to relationships and achievement compared to nurses without burnout. Intention to rotate was another factor examined in this country, where 56% of nurses had a high intention to rotate, according to Zhang et al.. In turn, Qi et al. showed that mentally passive sedentary time was associated with psychological distress and insomnia. Regarding quality of life, most Chinese HCWs had a fair perception, which could be modified by night shifts, aerobic exercise, and personality traits, as reported by Huang J. et al.. In the case of Bai et al., the importance of an appropriate leadership style in reducing burnout was noted.

In Korea, according to Kim et al., 28.0% of nurses had moderate depression, while 9.6% had severe depression, associated with high levels of interleukin-6, interleukin-8, and interleukin-18, and low levels of interferon-gamma.

In another study by Yacoubian et al. on medical students in Lebanon, prevalence rates of high burnout were 37.2% for disengagement and 51.1% for exhaustion. Along the same lines, a study conducted by Thu Pham et al. on HCWs in Vietnam reported a prevalence of symptoms of depression, anxiety, and stress of 19.2%, 24.7%, and 13.9%, respectively. Factors such as shift work during the pandemic, caring for patients with COVID-19, and staff health status were associated with mental health problems. In Qatar, according to the study by Al-Qudimat et al., psychological, social, and workplace effects were shown to be significantly related to marital status, career, and hospital setting, while dealing with COVID-19 patients, level of education, and working hours were related to clinical safety.

In Turkey, Sarigül et al. assessed the relationships between general job stress, suicidal ideation, hopelessness, and job satisfaction.

In Australia, a study was carried out on frontline HCWs during the COVID-19 pandemic (Omicron wave) by En Chyi Lee et al.. It was found that 18.1% were identified as likely to have a mental health condition, and a further 15.3% were identified as having low wellbeing, with concerns about COVID-19 infection, relational stress, and younger age as risk factors.

A study carried out in four Latin American countries by Bonilla-Asalde et al. showed that the greater the fear of COVID-19, the greater the preventive behavior of HCWs toward COVID-19 infection. In Peru, a study on nurses was carried out by Soriano-Vázquez et al., where a positive relationship was found between emotional intelligence, conflict management, and job satisfaction. Another study on nurses by Morales-García et al. was conducted, which found that work engagement played a key mediating role between depression, self-efficacy, job performance, and life satisfaction. Meanwhile, in Brazil, Spröesser Alonso et al. found that 72.6% of the study participants experienced psychological distress.

A systematic review by Rizzo et al. found that nurses' burnout scores did not differ significantly before and during the pandemic. In the study by Yasin et al., 69.6% of nurses were satisfied with the personal, environmental, and psychological factors influencing their job satisfaction during the COVID-19 pandemic. Finally, García-Iglesias et al. identified several factors influencing mental health and sickness presenteeism during the COVID-19 pandemic, such as factors related to mental health, individual factors, factors related to the situation caused by COVID-19, and factors derived from working conditions.

Therefore, the mental health of HCWs can be affected in many ways: with symptoms of anxiety, depression, burnout or fear, poor wellbeing, mental and physical fatigue, low perception of health-related quality of life and self-perceived health, favoring practices such as presenteeism, poor work performance, and low job satisfaction, among others.

The conclusions drawn from the studies included in this Research Topic can be summarized as follows:

- The COVID-19 pandemic caused high stress among HCWs, although burnout scores did not differ significantly before, vs. during the pandemic.
- In addition to work-related factors, other social, individual, and organizational factors can influence the mental health and wellbeing of HCWs.
- Factors such as night shifts, working hours, caring for patients with COVID-19, staff health status, aerobic exercise conditioning, and personality traits may affect the mental health of HCWs.
- Psychological support for health center workers in responding to future epidemics would improve their mental health.

## Author contributions

JG-I: Conceptualization, Data curation, Formal analysis, Funding acquisition, Investigation, Methodology, Project administration, Resources, Software, Supervision, Validation, Visualization, Writing – original draft, Writing – review & editing. MY: Conceptualization, Data curation, Formal analysis, Funding acquisition, Investigation, Methodology, Project administration, Resources, Software, Supervision, Validation, Visualization, Writing – original draft, Writing – review & editing. JG-S: Conceptualization, Data curation, Formal analysis, Funding acquisition, Investigation, Methodology, Project administration, Resources, Software, Supervision, Validation, Visualization, Writing – original draft, Writing – review & editing. Y-SG: Conceptualization, Data curation, Formal analysis, Funding acquisition, Investigation, Methodology, Project administration, Resources, Software, Supervision, Validation, Visualization, Writing – original draft, Writing – review & editing.
